# Aquaculture‐driven evolution of the salmon louse mtDNA genome

**DOI:** 10.1111/eva.13572

**Published:** 2023-06-28

**Authors:** Karoline Hasti Rutle, Rasmus Skern‐Mauritzen, Frank Nilsen, Alejandro Mateos‐Rivera, Anne Grete Sørvik Eide, Eeva Jansson, María Quintela, Francois Besnier, Fernando Allyon, Helene Børretzen Fjørtoft, Kevin Alan Glover

**Affiliations:** ^1^ Institute of Marine Research Bergen Norway; ^2^ Department of Biological Sciences University of Bergen Bergen Norway; ^3^ Department of Biological Sciences in Aalesund Norwegian University of Science and Technology Aalesund Norway

**Keywords:** aquaculture, deltamethrin, environmental impact, *Lepeophtheirus salmonis*, welfare

## Abstract

Resistance toward the antiparasitic pyrethroid, deltamethrin, is reported in the Atlantic salmon louse (*Lepeophtheirus salmonis salmonis*), a persistent ectoparasite of farmed and wild salmonids. The resistance mechanism is linked to mitochondrial DNA (mtDNA), where genetic markers for resistance have been identified. Here, we investigated how widespread pyrethroid use in aquaculture may have influenced mtDNA variation in lice, and the dispersion of resistant haplotypes across the North Atlantic, using historical (2000–2002 “pre‐resistance”) and contemporary (2014–2017 “post‐resistance”) samples. To study this, we sequenced ATPase 6 and cytochrome b, genotyped two genetic markers for deltamethrin resistance, and genotyped microsatellites as “neutral” controls of potential population bottlenecks. Overall, we observed a modest reduction in mtDNA diversity in the period 2000–2017, but no reduction in microsatellite variation was observed. The reduction in mtDNA variation was especially distinct in two of the contemporary samples, fixed for one and two haplotypes, respectively. By contrast, all historical samples consisted of close to one mtDNA haplotype per individual*.* No population genetic structure was detected among the historical samples for mtDNA nor microsatellites. By contrast, significant population genetic differentiation was observed for mtDNA among some of the contemporary samples. However, the observed population genetic structure was tightly linked with the pattern of deltamethrin resistance, and we therefore conclude that it primarily reflects the transient mosaic of pyrethroid usage in time and space. Two historically undetected mtDNA haplotypes dominated in the contemporary samples, both of which were linked to deltamethrin resistance, demonstrating primarily two origins of deltamethrin resistance in the North Atlantic. Collectively, these data demonstrate that the widespread use of pyrethroids in commercial aquaculture has substantially altered the patterns of mtDNA diversity in lice across the North Atlantic, and that long‐distance dispersion of resistance is rapid due to high level of genetic connectivity that is observed in this species.

## 1 INTRODUCTION

Pests and the evolution of pesticide resistance are regarded among the major challenges that global food production faces. Understanding both resistance development and dispersal are important to provide better management strategies and prolong the efficacy of pesticides (Clark & Yamaguchi, 2002). The ectoparasitic salmon louse *Lepeophtheirus salmonis* has developed widespread resistance against the most used pesticides and has consequently become a persistent challenge to environmentally sustainable salmon aquaculture (Taranger et al., 2015).

The salmon louse is an obligate parasite of salmonid fishes and belongs to the Caligidae family of copepods (Boxaspen, 2006). The species exists as two genetically distinct subspecies residing in the Atlantic and Pacific (Skern‐Mauritzen et al., 2014). Their life cycle comprises eight developmental stages (Hamre et al., 2013). In the first two lecithotrophic planktonic larvae stages (Nauplius I and II) and the infective copepodid stage, salmon lice drift with the ocean currents. After attaching to a host, they molt into the chalimus I and II, pre‐adult I, pre‐adult II, and reproductive adult stages (Hamre et al., 2013). During the infective stages, lice feed on host epidermal cells, mucus, and blood (Boxaspen, 2006). For the host, this may give rise to osmoregulatory problems, stress, and wounds that increase the chance of infection by secondary pathogens (Birkeland & Jakobsen, 1997; Bjørn & Finstad, 1998; Fjelldal et al., 2020; Grimnes & Jakobsen, 1996; Nolan et al., 1999) that may ultimately result in death (Bjørn & Finstad, 1998; Fjelldal et al., 2020; Grimnes & Jakobsen, 1996).

During its larval planktonic stages, lice typically drift between 20 and 45 km before attaching to a host (Johnsen et al., 2016). Further dispersal depends on the type of host it attaches to (Johnsen et al., 2016), which in the Atlantic primarily means Atlantic salmon (*Salmo salar)* or anadromous sea trout (*Salmo trutta) and in some areas Arctic charr (Salvelinus alpinus)*. While sea trout typically undergo short‐distance migrations in the coastal zone (Berg & Berg, 1987), Atlantic salmon from the western and eastern Atlantic migrate long distances from their coastal rivers to common oceanic feeding grounds (Gilbey et al., 2021). Thus, while attached to a wild salmon host, lice may disperse and exchange genetic material over long distances.

Population genetic studies, based on different types of markers, have revealed a lack of genetic structure in lice throughout the Atlantic and have largely attributed these observations to the long‐distance exchange of genetic material while attached to a long‐migrating salmon host (Dixon et al., 2004; Glover et al., 2011; Tjensvoll et al., 2006; Todd et al., 2004; Nolan and Powell, 2009). Some studies, however, have reported genetic differences in samples of lice from the North Atlantic when “outlier loci,” that is, loci that are likely under selection, are included (Besnier et al., 2014; Glover et al., 2011; Jacobs et al., 2018). It is well documented that the analysis of loci under selection often identifies population genetic structure in highly marine species where presumed neutral markers often fail to reveal genetic structure (Breistein et al., 2022; Han et al., 2020; Quintela et al., 2020; Russello et al., 2012). In an extensive population genomic study on lice, multiple outlier loci involved in selective sweeps on chromosomes 1, 5, and 14 were identified (Besnier et al., 2014). However, these outliers did not reveal any geographically meaningful population structure. These authors identified two sweeps causatively linked to selection from the use of chemical delousing agents. Based on the distribution of resistance, it was therefore concluded by those authors that lice most likely consist of a single panmictic population in the North Atlantic, but that spatial and temporal patterns of pesticide usage may give rise to transient mosaic patterns of genetic differences among samples in markers linked to resistance.

A variety of delousing methods have been utilized to control lice on farmed salmonids (Overton et al., 2019). Chemical treatments have been the most important approach and include both orally administered drugs and bath treatments (Aaen et al., 2015). However, access to only a few pesticides has created a strong selection pressure driving recurrent developments of resistance (Aaen et al., 2015). Among the pesticides rendered, largely inefficient is the pyrethroid deltamethrin, which is applied through bathing and was introduced in Norway in 1994 (Aaen et al., 2015). Several farms had reported loss in treatment efficiency (Aaen et al., 2015) and the peak of deltamethrin resistance measured by bioassays was reported in 2016 and 2017 (Jensen et al., 2020).

Several studies have demonstrated maternal inheritance of deltamethrin resistance in salmon lice, and since mitochondrial inheritance is commonly regarded as exclusively maternal, it supports the reported mitochondrial linkage of resistance (Bakke et al., 2018; Carmona‐Antoñanzas et al., 2017; Giles et al., 1980; Tschesche et al., 2022). A study on resistant and sensitive strains of salmon lice collected in Norway revealed five mtDNA SNPs showing association with deltamethrin resistance (Nilsen & Espedal, 2015). Later on, when studying resistant and sensitive salmon lice collected in 2018–2019 in Scotland, three mtDNA haplotypes associated with deltamethrin resistance were identified (Tschesche et al., 2022). All resistant haplotypes shared one mutational SNP, T8600C located in the Cox1 gene, which was therefore suggested to be the causative marker for resistance (Tschesche et al., 2022).

Two recent studies have revealed almost uniform mtDNA haplotype composition among deltamethrin‐resistant salmon lice collected in farms in Scotland (Carmona‐Antoñanzas et al., 2017; Tschesche et al., 2022). This is presumably the result of selection driven by the pyrethroids deltamethrin and cypermethrin delousing agents. This pattern strongly contrasts the high number of mtDNA haplotypes observed in a study of salmon lice throughout the North Atlantic in the period 2000–2002, before extensive deltamethrin usage (Tjensvoll et al., 2006). These observations support earlier suggestions that aquaculture represents a major driver of the population dynamics of salmon lice (Fjørtoft et al., 2017, 2020, 2021) and can induce rapid evolutionary changes (Besnier et al., 2014; Dempster et al., 2021; Mennerat et al., 2017).

The present study was designed to address the emergence and dispersion of deltamethrin resistance across the North Atlantic, and to investigate how widespread selection for resistance has influenced mtDNA variation after two decades of extensive pyrethroid use. In order to address these aims, a collection of historical (year 2000–2002, “pre‐resistance”) and contemporary (year 2014–2017, “post‐resistance”) salmon lice sampled throughout the North Atlantic were assigned to resistant/non‐resistant status based on markers for pyrethroid resistance, and, sequenced for two mitochondrial genes: ATPase 6 (A6) and Cytochrome B (Cyt b). Our most important findings were: an overall reduction in mtDNA variation of salmon lice with time, in addition to an emergence of two haplotypes that were not present in any of the historical samples but now dominate in the North Atlantic, both of which are linked to the causative mutation for deltamethrin resistance.

## 2 MATERIALS AND METHODS

### 2.1 Samples and data sources

**FIGURE 1:**
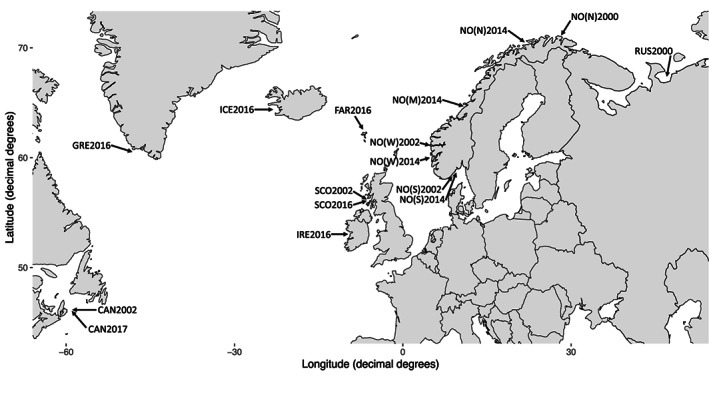
Map over sample locations for salmon lice collected in the North Atlantic. Sampling year is placed behind each locations shortened name. Map created using the R‐package ggplot2 (Wickham, 2016).

The present study is based on genetic data from 493 salmon lice collected throughout the North Atlantic, including 180 salmon lice collected between the years 2000 and 2002 (hereafter referred to as the historical samples—that is, before widespread pyrethroid resistance was reported) and 313 salmon lice collected between the years 2014 and 2017 (hereafter referred to as the contemporary samples—that is, after widespread pyrethroid resistance was reported) (Table 1, Figure 1). Both the historical and contemporary samples originate from larger sample collections used in multiple research projects (Besnier et al., 2014; Fjørtoft et al., 2017, 2020, 2021; Glover et al., 2011; Tjensvoll et al., 2006). Although some of the data presented here existed from previous studies, most of it was produced within the present study (Table 2).

**TABLE 1:** Overview of the historical and contemporary samples of salmon lice used in the present study.

Sampling location	Sampling year	Host species	*N*	Abbreviation
Historical				
Norway (Finnmark)	2000	Farmed salmon	30	NO(N)2000
Norway (Vestland)	2002	Farmed salmon	30*	NO(W)2002
Norway (Aust‐Agder)	2002	Wild seatrout	30	NO(S)2002
Scotland	2002	Farmed salmon	30	SCO2002
Russia	2000	Wild salmon	30*	RUS2000
Canada	2002	Farmed salmon	30	CAN2002
Contemporary				
Norway (Finnmark)	2014	Wild salmon	31	NO(N)2014
Norway (Trøndelag)	2014	Wild salmon	32	NO(M)2014
Norway (Vestland)	2014	Wild salmon	31	NO(W)2014
Norway (Aust‐Agder)	2014	Wild seatrout	31	NO(S)2014
Scotland	2016	Farmed salmon	32	SCO2016
Faroe Islands	2016	Farmed salmon	31	FAR2016
Iceland	2016	Wild salmon	31	ICE2016
Ireland	2016	Farmed salmon	31	IRE2016
Greenland	2016	Wild salmon	32	GRE2016
Canada	2017	Farmed salmon	31	CAN2017

*Note*: The table shows sampling locations with shortened names (abbreviation), sampling year, and the number of individuals (*N*).

* Microsatellite; sample size of 29 individuals for Vestland (2002), sample size of 32 for Russia (2000).

**TABLE 2:** Overview of the types of data used in the present study and their origins.

Sample	Microsatellites	mtDNA sequences	Pyrethroid patented marker (C14065T)	Pyrethroid SNP causative (T8600C)
Historical	Present study	Tjensvoll et al. (2006) (GenBank; accession numbers; A6: AY602407–AY602586; Cyt b: AY602223–AY602402)	Fjørtoft et al. (2021)	Tjensvoll et al. (2006) (GenBank; accession numbers; COX1: AY602587–AY602766)
Contemporary	Present study	Present study	Fjørtoft et al. (2021)	Present study

The data in this study include (i) the genotypes providing information on resistance to pyrethroids (deltamethrin), using two genetic markers for this. The first marker for deltamethrin resistance is the patented non‐causative genetic marker C14065T that has been associated with resistance (Nilsen & Espedal, 2015). The data for this marker were obtained from Fjørtoft et al. (2021). The second marker included the T8600C mutation that was recently discovered (Tschesche et al., 2022), and it is presumed to be causative for deltamethrin resistance. Data for this marker were acquired in the present study by genotyping all of the samples with this marker as described below; (ii) sequences for the mitochondrial genes A6 and Cyt b that were used to study spatial and temporal changes in mtDNA variation between the historical (Tjensvoll et al., 2006) and contemporary salmon lice samples; and (iii) genotypes at 15 microsatellite DNA loci. Microsatellites are highly effective in picking up strong demographic events such as inbreeding and strong population bottlenecks (Skaala et al., 2004; Skern‐Mauritzen et al., 2013). Microsatellites were therefore included in the present study to provide a presumed selectively neutral nDNA control for potential temporal or spatial patterns observed in the mtDNA variation.

### 2.2 Description of genetic analyses

The molecular analyses performed within the present study comprised mtDNA sequencing of the contemporary samples, genotyping microsatellites on the historical and contemporary samples, and genotyping the T8600C SNP on the contemporary samples. These procedures are described below. Molecular analyses associated with previously published data are found in the original references.

### 2.3 MtDNA amplification, sequencing, and data treatment

Contemporary samples were sequenced using primers targeting A6 and cytochrome b Cyt b (Tjensvoll et al., 2006). Both mtDNA genes were amplified in a 15‐μL PCR mix consisting of 1x Buffer, 2 mM MgCl_2_, 0.15 mM dNTP, 1 U GoTaq polymerase (Promega), 0.4 μM of each primer, and 3 μL of the template. PCR amplification was performed at 95°C; 35 cycles of 94°C for 30 s, 48°C for 30 s, 72°C for 1 min; and 72°C 7 min; and purified following the procedure described in Mateos‐Rivera et al. (2020), before being sent for sequencing in duplicates (one per each direction) to Eurofins Genomics.

Sequences were quality controlled and manually trimmed using Geneious Prime 2021.2.2 (https://www.geneious.com). Forward and reverse sequences were then assembled into contigs, and consensus sequences were aligned against the historical sequences using MUSCLE (Edgar, 2004) default settings in Geneious Prime 2021.2.2. All polymorphisms (i.e., SNPs) were quality checked by visual inspection of chromatograms.

### 2.4 Microsatellite genotyping

The historical and contemporary salmon lice samples were genotyped for 15 microsatellite DNA loci. Genotyping was conducted in three multiplex reactions consisting of the following markers: Multiplex one; *Lsa1STA1*, *Lsa1STA2*, *Lsa1STA4*, *Lsa1STA5*, *LsNUIG14* (Todd et al., 2004). Multiplex two; *Lsal103EUVC*, *Lsal109EUVC*, *Lsal110EUVC*, *Lsal111EUVC* (Messmer et al., 2011) and *NUIG9* (Nolan et al., 2000). Multiplex three; *Lsal104EUVC*, *Lsal105EUVC*, *Lsal106EUVC*, *Lsal108EUVC* (Messmer et al., 2011) and *Lsa1STA3* (Todd et al., 2004). With minor concentration adjustments, PCR and amplification conditions followed those described previously (Glover et al., 2011). Microsatellites were independently scored by two different people, followed by a routine check of the genotyping consistency as recommended by Pompanon et al. (2005). This was performed by reanalyzing 144 individuals. After independent scoring of the reanalyzed individuals, 489 genotypes spread across the 15 microsatellite loci were compared between first and second genotyping. A total of two inconsistencies were observed giving an overall allelic genotyping consistency rate of 99.8%. Due to challenges relating to DNA quality, a 50% threshold for allowed missing data per individual as a combined measure for all 15 markers was adopted. Therefore, the final microsatellite dataset consisted of genotypes for 365 lice displaying >50% genotyping coverage (other thresholds were initially tested, all giving highly similar results, data not presented).

### 2.5 Deltamethrin‐resistant SNP amplification, genotyping, and processing

Data for resistance status according to a patented non‐causative marker for pyrethroid resistance C14065T (Nilsen & Espedal, 2015) were obtained from Fjørtoft et al. (2021). The more recently discovered and presumed causative mutation for pyrethroid resistance, T8600C (Tschesche et al., 2022), was identified by genotyping in the present study on the contemporary samples. The historical lice samples used in the present study were checked for T8600C mutation in sequence data published previously by Tjensvoll et al. (2006) (Table 2) and were all found to be sensitive according to the T8600C marker.

Genotyping the T8600C marker for the contemporary samples was done by a 5´nuclease reverse transcriptase real‐time quantitative PCR TaqMan assay. Primers used were targeting the mutant SNP T8600C (accession number; LT630766) (Tschesche et al., 2022). Two‐step amplification with 45 cycles was conducted on Applied Biosystem 7500 real‐time PCR following the manufacture instructions (ThermoFisher). The salmon lice were genotyped as either resistant or sensitive for deltamethrin. Positive controls belonging to the historical samples with known genotypes (Genebank accession number: AY602587‐AY602589, AY602591, AY602596) were included, and resulted in expected sensitive genotypes for the T8600C genotyping.

### 2.6 Genetic variation

For the mitochondrial sequences, differences in genetic variation among samples were measured for the combined haplotypes (i.e., where the combined gene haplotypes for A6 and Cyt B represented a single mitochondrial haplotype) using standard molecular indices in the software DnaSP v6 (Rozas et al., 2017). To account for uneven sample sizes, a rarefaction method was used to quantify haplotype richness (HR; number of haplotypes per sample adjusted for minimum sample size) and private haplotype richness (PR; level of unique haplotypes per sample adjusted for the minimum sample size) in the software HP‐RARE v1.0 (Kalinowski, 2005). In the rarefaction analysis, the minimum sample size was 16 individuals when comparing the different subsamples and 180 individuals when comparing between the historical and the contemporary datasets. Measures of genetic variation were repeated with independent measures for the genes separately to account for the theoretical possibility for mitochondrial recombination in heteroplasmid individuals (see section 1.1 in Data S1).

For the microsatellite data, the software Arlequin v3.5.2.2 (Excoffier & Lischer, 2010) was used to summarize the number of alleles, the observed (*H*o) and expected (*H*e) heterozygosity, deviation against Hardy–Weinberg equilibrium (HWE), and the inbreeding coefficient (*F*_IS_). Allelic richness (A_R_; average number of alleles per sample adjusted for minimum sample size) and the number of private alleles (N_PA_; number of unique alleles per sample adjusted for minimum sample size) for each sample. The number of private alleles observed, when comparing between the historical and contemporary dataset was calculated using microsatellite analyzer (MSA) (Dieringer & Schlötterer, 2003). For allelic richness, the minimum sample size was set to 20 individuals, and samples below the minimum sample size were excluded from the analysis of allelic richness. FSTAT (Goudet, 1995) was used in the measure of allelic richness when comparing between the historical and contemporary dataset, with a minimum sample size set to 144 individuals.

The difference in genetic variation between the historical and contemporary samples was tested with a two‐sided *t*‐test using the *t.test* function from the stats package in R studio v1.4.1106 (R Core Team, 2021). Differences were considered statistically significant if *p* < 0.05.

### 2.7 Population genetic structure

Although the analysis of population genetic structure was not a major part of the present study, temporal and spatial changes in population genetic structure for the combined mtDNA haplotypes across both of these genes, and for the 15 microsatellites, were measured using analysis of pairwise genetic differentiation (*F*_ST_ for microsatellites and *Φ*_ST_ for haplotypes (Weir & Cockerham, 1984)) implemented in Arlequin v3.5.2.2 (Excoffier & Lischer, 2010). Pairwise genetic differentiation for the microsatellites was measured using F‐statistics to compare differentiation in allelic frequencies between samples (*F*_ST_). For the mitochondrial genes, pairwise genetic differentiation was measured using F‐statistics to calculate the genetic distance between haplotypes (*Φ*_ST_).

Population genetic structure was also investigated for each mtDNA gene separately (see section 1.2 in Data S1). The significance of the calculated statistics measured by the analysis of pairwise differences was tested using the permutation approach in Arlequin v3.5.2.2 (Excoffier & Lischer, 2010). The significance level of 0.05 and 10,000 permutations was set to test the null hypothesis of a panmictic population of North Atlantic salmon lice for both mtDNA and microsatellites. The resulting p‐values were corrected using the Holm–Bonferroni sequential correction method (Gaetano, 2013).

### 2.8 Haplotype distribution

To visualize the distribution of haplotypes in all historical and contemporary samples, networks were generated using sequence data for A6 and Cyt b combined, with the software PopART (Leigh & Bryant, 2015). Haplotype networks were also generated for each mtDNA gene separately (section 1.3 in Data S1). All haplotype networks were constructed using the medium joint network approach, with the search for medium vectors set to a low minimum (ε being 0) to ease the presentation of the network.

Furthermore, haplotype networks based on resistance data were generated for the contemporary samples. Deltamethrin resistance based on the C14065T marker (Nilsen & Espedal, 2015) as well as the T8600C marker (Tschesche et al., 2022) were used separately to identify mtDNA haplotypes as being either resistant or sensitive.

## 3 RESULTS

### 3.1 Data description

A total of 249 salmon lice collected from 10 locations in the North Atlantic between 2014 and 2017 (contemporary samples) were successfully sequenced for both ATPase 6 (A6, 520 bp sequenced) and cytochrome b (Cyt b, 693 bp sequenced), resulting in a combined sequence length of 1213 bp. These sequences were aligned to 180 salmon lice mtDNA sequences downloaded from GenBank [A6: AY602407‐AY602586, Cyt b: AY602223‐AY602402], originating from six locations in the North Atlantic in the period 2000–2002 (historical samples) (Tjensvoll et al., 2006).

Of the 249 contemporary salmon lice successfully sequenced for A6 and Cyt b, 237 were also successfully genotyped for the T8600C genetic marker to check resistant status for deltamethrin. The 180 historical salmon lice were checked for resistance status according to the T8600C genetic marker by studying the COX‐1 gene sequences presented in by Tjensvoll et al., 2006 (Table 2.). Deltamethrin‐resistant status on the salmon lice used in this study according to the non‐causative C14065T marker was genotyped by Fjørtoft et al. (2021). A total of 196 salmon lice from the contemporary sample and 169 salmon lice from the historical samples were successfully genotyped for 15 microsatellite loci.

### 3.2 Pyrethroid resistance

None of the lice representing the historical samples were pyrethroid resistant according to either the C14065T or the T8600C markers. In the contemporary samples, 39% of the lice were identified as resistant from at least one of the two markers, although this differed by marker (Table 3). According to C14065T, the highest percentages of deltamethrin resistance were observed in the SCO2016 sample (47%; Table 3), while no evidence of resistance was found in the CAN2017 and FAR2016 contemporary samples. By contrast, according to T8600C, the SCO2016 and FAR2016 sample had the highest percentage of deltamethrin resistance (100%; Table 3).

**TABLE 3:** Summary of mtDNA haplotype diversity (cytochrome b and ATPase 6 combined) and microsatellite diversity for the historical and contemporary samples.

mtDNA									Microsatellites							
Sample	Host species	Pyrethroid resistance (C14065T) (%)	Pyrethroid resistance (T8600C) (%)	*N*	*N*_H_	*H*_R_	*P*_R_	*N*_SNP_	*N*	*N*_A_	*N*_PA_	*H*_O_ (sd)	*H*_E_ (sd)	HWE	*F*_IS_	*A*_R_
Historical																
NO(N)2000	FS	0	0	30	30	23.46	19.87	65	21	89	0	0.494 (0.350)	0.477 (0.335)	0	−0.039 *p* = 0.873	5.99
NO(W)2002	FS	0	0	30	28	22.36	19.22	57	29	109	3	0.501 (0.310)	0.521 (0.323)	0	0.037 *p* = 0.115	6.35
NO(S)2002	WT	0	0	30	30	23.59	17.82	74	30	111	0	0.498 (0.343)	0.510 (0.315)	2	0.012 *p* = 0.356	6.47
SCO2002	FS	0	0	30	28	22.36	20.14	66	30	112	4	0.487 (0.301)	0.523 (0.313)	2	0.064 *p* = 0.020	6.55
RUS2000	WS	0	0	30	29	22.98	18.64	64	29	111	2	0.451 (0.340)	0.485 (0.333)	1	0.065 *p* = 0.017	6.53
CAN2002	FS	0	0	30	24	19.77	14.90	60	30	113	4	0.514 (0.311)	0.530 (0.313)	1	0.029 *p* = 0.184	6.64
Contemporary																
NO(N)2014	WS	21	20*	29	24	19.48	13.99	60	19	97	4	0.507 (0.349)	0.517 (0.333)	3	0.000 *p* = 0.530	n.a.
NO(M)2014	WS	24	28	25	22	19.38	15.06	54	23	93	6	0.460 (0.311)	0.498 (0.317)	3	−0.078 *p* = 0.977	6.63
NO(W)2014	WS	40	39*	30	19	15.16	11.07	53	24	102	3	0.484 (0.336)	0.493 (0.322)	1	−0.011 *p* = 0.664	6.45
NO(S)2014	WT	0	6*	18	18	17.83	12.28	43	18	81	1	0.583 (0.324)	0.642 (0.253)	1	0.045 *p* = 0.152	n.a.
SCO2016	FS	47	100*	32	2	2.00	0.00	13	17	87	1	0.453 (0.323)	0.495 (0.302)	2	0.076 *p* = 0.036	n.a.
FAR2016	FS	0	100	16	1	1.00	0.00	0	14	76	0	0.562 (0.334)	0.600 (0.287)	1	−0.002 *p* = 0.545	n.a.
ICE2016	WS	13	14*	23	21	19.32	13.10	63	23	91	2	0.522 (0.380)	0.518 (0.335)	1	−0.122 *p* = 1.000	5.58
IRE2016	FS	11	56*	28	16	13.49	7.75	40	12	87	4	0.589 (0.325)	0.584 (0.319)	0	−0.018 *p* = 0.711	n.a.
GRE2016	WS	16	26	19	16	15.70	11.99	54	21	93	3	0.520 (0.349)	0.524 (0.321)	1	−0.048 *p* = 0.916	6.28
CAN2017	FS	0	0	29	22	18.22	10.08	59	25	91	4	0.475 (0.303)	0.520 (0.293)	1	0.025 *p* = 0.257	5.99
Historical		0	0	180	158	158.00	140.08	181	169	170	27	0.473 (0.326)	0.491 (0.328)	3	0.031 *p* = 0.007	10.18
Contemporary		19	39	249	133	123.86	105.95	168	196	180	37	0.466 (0.344)	0.487 (0.330)	1	−0.024 *p* = 0.975	10.63

*Note*: The table shows the host species for the collected salmon lice (WS—wild salmon, WT—wild seatrout, FS—farmed salmon), the percentage of pyrethroid resistance, sample size (*N*) for mtDNA, the observed number of haplotypes (*N*_H_), haplotype richness (*H*_R_), private haplotype richness (*P*_R_), the number of SNPs (*N*_SNP_) in each sample for the mtDNA. For the microsatellites, the table shows the sample size (*N*), total number of alleles across 15 microsatellites (*N*_A_), the number of private alleles (*N*_PA_), observed heterozygosity (H_O_) with standard deviation in brackets, expected heterozygosity (*H*_E_) with standard deviation in brackets, the number of loci deviating from the Hardy–Weinberg equation (HWE), the Inbreeding coefficient (*F*_IS_), and allelic richness (*A*_R_) adjusted for a minimum sample size.

* Resistance data based on one individual less than the sample size (*N*). NO(N)2014; resistance data based on 25 individuals. NO(W)2014; resistance data based on 28 individuals.

### 3.3 Genetic variation in mtDNA sequences and microsatellites

Standard indices of genetic diversity within each of the historical and contemporary samples were calculated using data from the two mtDNA genes combined into haplotypes (Table 3), for the two mtDNA genes separately (section 1.1 in Data S1; Tables S1 and S2), and for the 15 microsatellite loci (Table 3).

For the two mtDNA genes combined, 249 salmon lice from the contemporary samples displayed 133 unique haplotypes (Table 3). The contemporary salmon lice consisted of, in total, 1045 monomorphic nucleotide sites and 168 polymorphic variable sites, divided into 85 parsimony‐informative (P) and 83 singletons sites. The 180 historical salmon lice mtDNA sequences included 158 unique haplotypes, 1032 monomorphic nucleotide sites, and 181 polymorphic variable sites separated into 94 parsimony‐informative and 87 singleton variable sites.

Haplotype richness (HR) was significantly higher in the historical (mean = 22.42, SD = 1.39) than in the contemporary samples (mean = 14.16, SD = 6.97) (two‐sample *t*‐test: HR: *t* (10.16) = − 3.62, *p* = 0.004). This was strongly driven by the contemporary SCO2016 and FAR2016 samples displaying drastically reduced haplotype richness (Table 3). Nevertheless, haplotype richness decreased between the historical (mean = 22.42, SD = 1.39) and contemporary samples (mean = 17.32, SD = 2.26), even when excluding the SCO2016 and FAR2016 samples (two‐sample *t*‐test: HR: *t* (11.69) = − 5.18, *p* = 0.0002). There was also a decrease in haplotype richness in the contemporary samples (mean = 17.81, SD = 1.94) compared to the historical samples (mean = 22.42, SD = 1.39) when excluding contemporary samples collected from farmed hosts (two‐sample *t*‐test: HR: *t* (9.07) = −4.70, *p* = 0.0001). Other estimates of haplotype diversity and richness were consistent with these results (Table 3).

Across the 15 pooled microsatellites, a total of 180 and 169 alleles were observed for the contemporary and historical samples, respectively (Table 3). There was a higher allelic richness for the pooled contemporary samples than for the pooled historical samples when adjusting for sample size of 144 individuals. No significant difference in allelic richness between the historical (mean = 6.42, SD = 0.23) and the contemporary (mean = 6.19, SD = 0.41) samples was observed when adjusted for a minimum sample of 20 individuals (two‐sample *t*‐test: AR: *t* (6.05) = −1.14, *p* = 0.299). Notably, even in the two samples displaying 100% pyrethroid resistance and just one or two mtDNA haplotypes (haplotypes 165 and 174), no clear evidence of loss of microsatellite alleles was detected.

### 3.4 Population genetic structure

To investigate spatial and temporal genetic structure among samples, a full matrix of pairwise comparisons were conducted using the two mtDNA genes combined (Table 4a), the mtDNA genes separately (section 1.2 in Data S1; Tables S3 and S4), and the 15 microsatellite loci (Table 4b).

**TABLE 4:** Pairwise genetic differences among historical and contemporary samples of lice using: (a) mtDNA cytochrome b and ATPase 6 (Φ_ST_ in the lower diagonal, *p*‐value in the upper right diagonal), (b) 15 microsatellites (*F*_ST_ in the lower diagonal, Holm–Bonferroni sequential corrected *p*‐value in the upper right diagonal).

(a)	Historical						Contemporary									
	NO(N)2000	NO(W)2002	NO(S)2002	SCO2002	RUS2000	CAN2002	NO(N)2014	NO(M)2014	NO(W)2014	NO(S)2014	SCO2016	FAR2016	ICE2016	IRE2016	GRE2016	CAN2017
Historical																
NO(N)2000	*	1.000	1.000	1.000	1.000	1.000	1.000	1.000	0.099	1.000	**0.000**	**0.000**	1.000	0.588	1.000	1.000
NO(W)2002	0.005	*	1.000	1.000	1.000	1.000	1.000	1.000	0.440	1.000	**0.000**	**0.000**	1.000	0.648	1.000	1.000
NO(S)2002	0.008	0.000	*	1.000	1.000	1.000	1.000	1.000	1.000	1.000	**0.000**	**0.000**	1.000	1.000	1.000	1.000
SCO2002	0.016	0.000	0.000	*	1.000	1.000	1.000	1.000	0.190	1.000	**0.000**	**0.000**	1.000	0.588	1.000	1.000
RUS2000	0.006	0.000	0.007	0.000	*	1.000	1.000	1.000	0.190	1.000	**0.000**	**0.000**	1.000	1.000	1.000	1.000
CAN2002	0.000	0.015	0.002	0.017	0.013	*	1.000	1.000	0.099	1.000	**0.000**	**0.000**	1.000	0.360	1.000	1.000
Contemporary																
NO(N)2014	0.010	0.013	0.000	0.019	0.014	0.008	*	1.000	1.000	1.000	0.190	**0.000**	1.000	1.000	1.000	1.000
NO(M)2014	0.021	0.001	0.000	0.012	0.006	0.022	0.000	*	1.000	1.000	0.440	**0.000**	1.000	1.000	1.000	1.000
NO(W)2014	0.104	0.071	0.041	0.079	0.087	0.096	0.026	0.007	*	1.000	0.276	**0.000**	0.588	0.510	1.000	0.099
NO(S)2014	0.000	0.000	0.000	0.000	0.000	0.003	0.000	0.000	0.052	*	0.276	**0.000**	1.000	1.000	1.000	1.000
SCO2016	0.197	0.203	0.177	0.197	0.180	0.205	0.139	0.124	0.158	0.184	*	0.440	0.099	1.000	0.960	**0.000**
FAR2016	0.429	0.483	0.438	0.454	0.437	0.442	0.429	0.453	0.541	0.523	0.308	*	**0.000**	**0.000**	**0.000**	**0.000**
ICE2016	0.000	0.004	0.000	0.014	0.013	0.003	0.000	0.001	0.077	0.000	0.160	0.397	*	1.000	1.000	1.000
IRE2016	0.059	0.062	0.051	0.060	0.044	0.071	0.041	0.028	0.095	0.037	0.028	0.297	0.040	*	1.000	0.360
GRE2016	0.011	0.000	0.000	0.011	0.008	0.021	0.000	0.000	0.005	0.000	0.130	0.475	0.000	0.027	*	1.000
CAN2017	0.000	0.004	0.000	0.019	0.006	0.000	0.009	0.019	0.099	0.000	0.219	0.469	0.000	0.073	0.015	*

(b)	Historical						Contemporary									
	NO(N)2000	NO(W)2002	NO(S)2002	SCO2002	RUS2000	CAN2002	NO(N)2014	NO(M)2014	NO(W)2014	NO(S)2014	SCO2016	FAR2016	ICE2016	IRE2016	GRE2016	CAN2017
Historical																
NO(N)2000	*	1.000	1.000	1.000	1.000	1.000	1.000	1.000	1.000	1.000	1.000	1.000	1.000	1.000	1.000	1.000
NO(W)2002	0.000	*	1.000	1.000	1.000	1.000	1.000	1.000	1.000	1.000	1.000	1.000	1.000	1.000	1.000	1.000
NO(S)2002	0.000	0.000	*	1.000	1.000	1.000	1.000	1.000	1.000	1.000	1.000	1.000	1.000	1.000	1.000	1.000
SCO2002	0.006	0.000	0.003	*	1.000	1.000	1.000	1.000	1.000	1.000	1.000	1.000	1.000	1.000	1.000	1.000
RUS2000	0.002	0.000	0.000	0.000	*	1.000	1.000	1.000	1.000	1.000	1.000	1.000	1.000	1.000	1.000	1.000
CAN2002	0.007	0.000	0.001	0.000	0.000	*	1.000	1.000	1.000	1.000	1.000	1.000	1.000	1.000	1.000	1.000
Contemporary																
NO(N)2014	0.005	0.000	0.000	0.000	0.000	0.003	*	1.000	1.000	1.000	1.000	1.000	1.000	1.000	1.000	1.000
NO(M)2014	0.000	0.000	0.000	0.000	0.000	0.000	0.000	*	1.000	1.000	1.000	1.000	1.000	1.000	1.000	1.000
NO(W)2014	0.005	0.000	0.000	0.001	0.000	0.001	0.000	0.000	*	1.000	1.000	1.000	1.000	1.000	1.000	1.000
NO(S)2014	0.000	0.000	0.000	0.000	0.000	0.000	0.000	0.000	0.000	*	1.000	1.000	1.000	1.000	1.000	1.000
SCO2016	0.000	0.000	0.004	0.006	0.001	0.010	0.003	0.000	0.001	0.000	*	1.000	1.000	1.000	1.000	1.000
FAR2016	0.005	0.000	0.000	0.000	0.000	0.000	0.000	0.000	0.000	0.000	0.003	*	1.000	1.000	1.000	1.000
ICE2016	0.000	0.000	0.000	0.000	0.000	0.000	0.000	0.000	0.000	0.000	0.000	0.000	*	1.000	1.000	1.000
IRE2016	0.004	0.000	0.000	0.000	0.000	0.000	0.001	0.000	0.000	0.000	0.001	0.000	0.000	*	1.000	1.000
GRE2016	0.000	0.000	0.000	0.000	0.000	0.000	0.000	0.000	0.000	0.000	0.000	0.000	0.000	0.000	*	1.000
CAN2017	0.006	0.000	0.001	0.002	0.004	0.000	0.000	0.000	0.003	0.000	0.004	0.010	0.000	0.003	0.000	*

*Note*: Significant values <0.05 are highlighted in bold. The color gradient shows the level of differentiation as suggested by Wright (1978). Green being low differentiation (<0.05), yellow intermediate (0.05–0.15), and red high differentiation (>0.15).

For the analyses using the combined mtDNA sequences, no significant genetic differentiation was observed among the historical samples (Table 4a). However, among the contemporary samples, significant population genetic differentiation was observed. In particular, the FAR2016 and SCO2016 samples deviated significantly from each other and from all other samples both compared to the historical and contemporary samples. It is important to note that these samples comprised only one (FAR2016) and two haplotypes (SCO2016), respectively, which is the reason for the substantial genetic differentiation observed. By contrast, no genetic differentiation was observed among any historical or contemporary samples based on the microsatellite data (Table 4b).

### 3.5 Haplotype distribution

**FIGURE 2:**
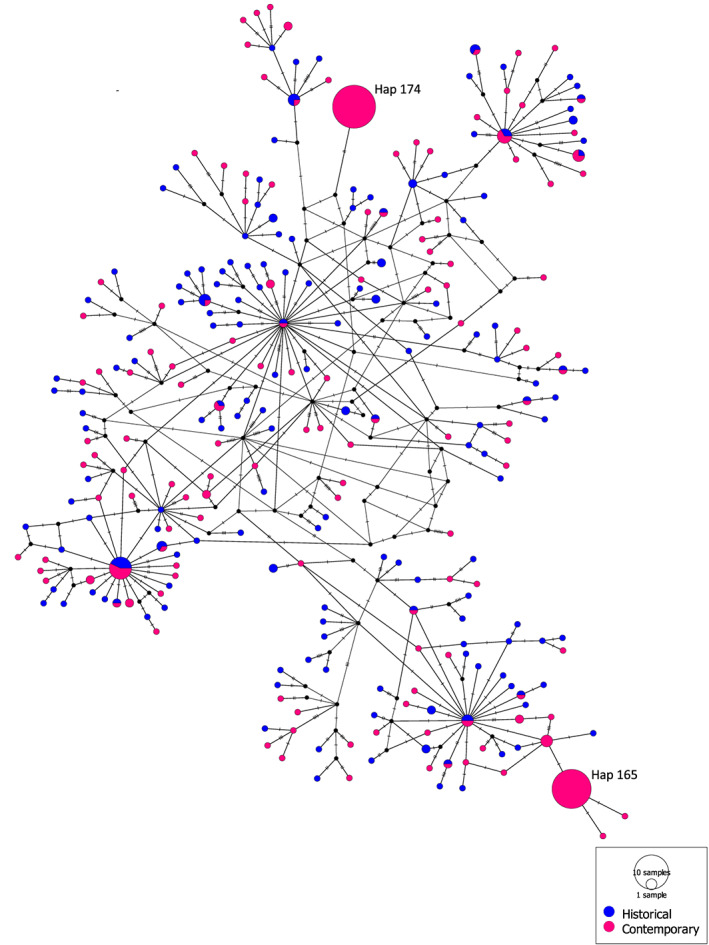
Haplotype network of the historical (blue) and contemporary (pink) samples of salmon lice (haplotypes created across cytochrome b and ATPase 6). Note the emergence of two haplotypes within the contemporary sample (Hap 165 and Hap 174). Nodes represent one haplotype. The size and the color within each node correspond to the number of sequences per group that shares the haplotype. The number of homologous nucleotide sites where two haplotypes differ is represented by the number of parallel lines. 0–1 parallel line represents one mutational difference as does a black dot.

**FIGURE 3:**
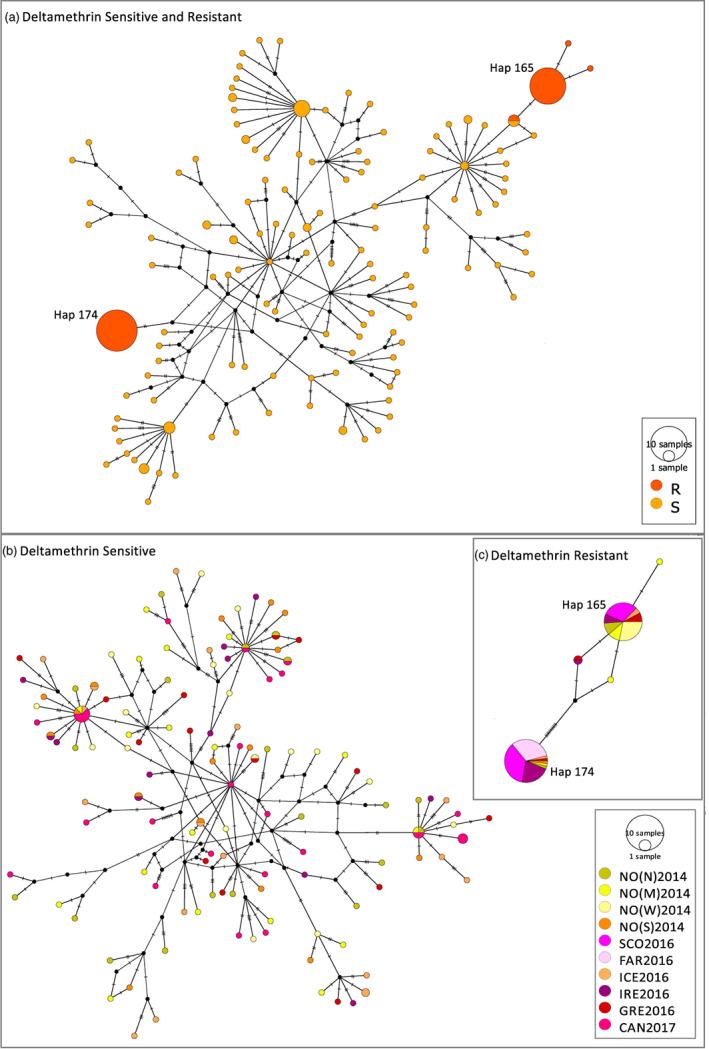
Haplotype network of the contemporary samples of salmon lice (haplotypes created across cytochrome b and ATPase 6). (a) Network separated into sensitive (S) and resistant individuals (R) for deltamethrin based on the T8600C genetic marker. (b) Network of sensitive (S) individuals and (c) network of resistant (R) individuals for deltamethrin based on the T8600C genetic marker separated by sample location. The size and the color within the nodes correspond to the number of sequences per group that share a haplotype. The number of homologous nucleotide sites where two haplotypes differ is represented by the number of parallel lines. 0–1 parallel line represents one mutational difference as does one black dot. The figure shows the emergence of two haplotypes (Hap 174 and Hap 165), with the most pyrethroid‐resistant individuals displaying Hap 165 and Hap 174.

**FIGURE 4:**
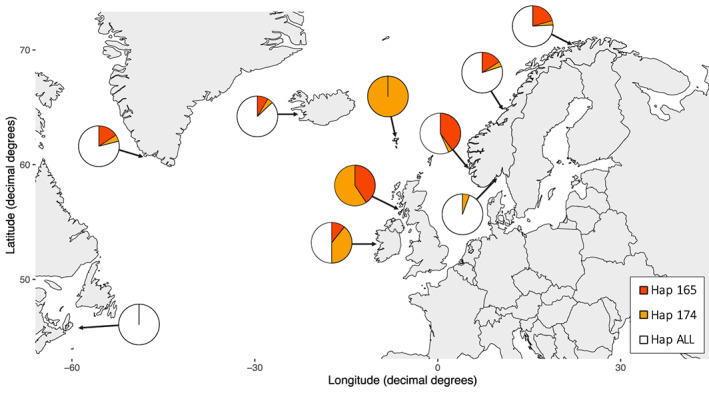
Map of North Atlantic, showing the distribution of the haplotypes emerging in the contemporary samples of lice across cytochrome b and ATPase 6 (Hap 165 and Hap 174), relative to all other haplotypes (Hap ALL). Neither of these haplotypes, both of which are linked with the genetic marker causatively associated with pyrethroid resistance, were present in any of the historical samples. This demonstrates a minimum of two independent origins of pyrethroid resistance in lice in the North Atlantic.

Haplotype networks were generated for the two mtDNA genes combined (Figures 2, 3, 4) and separately (section 1.3 in Data S1; Figures S2–S7) using the Medium Joint Network approach. The most striking observation from the networks is that haplotypes 165 and 174 were not observed in the historical samples but emerged as the two most frequent haplotypes in the contemporary samples (Figure 2). Of the 249 lice in the contemporary sample, 43 and 52 individuals displayed haplotypes 165 and 174, respectively (Figure 2). In the contemporary sample, deltamethrin‐resistant salmon lice according to both the C14065T and the T8600C genetic marker were only found among five haplotypes (section 1.3 in Data S1; Figure S8c and Figure 3).

According to T8600C, which is regarded as the causative mutation for pyrethroid resistance, almost all individuals displaying haplotypes 165 and 174 were resistant (Figure 3). By contrast, according to C14065T, which is a non‐causative marker for resistance, the majority of individuals that were of haplotype 165 were resistant while the majority of individuals that were of haplotype 174 were marked as sensitive (section 1.3 in Data S1; Figure S8c). Haplotype 165 was represented in all samples except NO(S)2014, FAR2016, and CAN2017 and haplotype 174 was represented in all samples except CAN2017 and NO(W)2014 (Figure 3b).

According to the T8600C marker, all lice found in the SCO2016 and FAR2016 samples were resistant (Table 3). By contrast, the highest percentage of deltamethrin‐resistant salmon lice identified by the C14065T marker was 47% found in the SCO2016 sample (Table 3). The SCO2016 sample only included haplotypes 165 and 174 (Figure 3b). According to the C14065T marker, 12 of the 13 SCO2016 individuals that were of haplotype 165 were identified as resistant, whereas 16 of the 19 SCO2016 salmon lice that were of haplotype 174 were suggested to be sensitive (section 1.3 in Data S1; Figure S8a,b). All lice within the FAR2016 sample were of haplotype 174 (Figure 3b), and notably, all were resistant to deltamethrin based on the T8600C marker (Figure 3b). By contrast, all were susceptible to deltamethrin based on the C14065T marker (section 1.3 in Data S1; Figure S8a).

Haplotype 174 dominated in lice from the Faroe Islands but was also well represented in Scotland and Ireland (Figure 4). Haplotype 165 was predominantly found along the Norwegian coastline, except for the southern–eastern part of Norway. At all sample locations, either both or one of the emerging haplotypes (haplotype 165 and haplotype 174) were found, except for the sample collected on the eastern coast of Canada (CAN2017).

## 4 DISCUSSION

Our analyses using an amphi‐Atlantic set of historical (pre‐resistance) and contemporary (post‐resistance) samples of salmon lice show: (1) an overall reduction in mtDNA variation with time, (2) the emergence of two new haplotypes linked to the causative mutation for deltamethrin resistance that now dominate in the North Atlantic, and (3) no evidence of population genetic structure throughout the North Atlantic in genetic markers not associated with chemical resistance. Based on these results, we conclude that widespread use of deltamethrin in commercial fish farms has caused changes in the salmon louse mtDNA genome, leading to widespread dispersal of resistance from a minimum of two independent sources. We also suggest, based on these results and work from previous studies, that when the temporal and spatial mosaic of pesticide use is accounted for, this species displays little evidence of population genetic structure throughout the North Atlantic.

### 4.1 Temporal changes in mtDNA variation

Historically, very high mtDNA variation has been observed in lice (Tjensvoll et al., 2006), as illustrated by the high proportion of singleton haplotypes in the historical samples analyzed here. We observed a modest but nevertheless significant reduction in mtDNA variation in the contemporary samples, which was still observed after excluding the contemporary samples collected from farmed hosts. By contrast, no change in microsatellite diversity was observed in the contemporary samples, not even in the samples displaying 100% resistance and just one or two mtDNA haplotypes. Microsatellites are very sensitive in picking up inbreeding and population bottlenecks (Skaala et al., 2004; Skern‐Mauritzen et al., 2013). Therefore, these data collectively demonstrate that the observed loss in mtDNA diversity is caused by resistance selection, and not by a demographic event such as a population collapse or bottleneck. In turn, this indicates that selection in one hereditome compartment, (i.e., a carrier of heritable information) (Skern‐Mauritzen & Mikkelsen, 2021) such as the mitochondrial genome, does not necessarily leave an imprint on other hereditome compartments, for example, the nuclear genome.

The observed reduction in mtDNA variation was most evident in the contemporary samples collected from farmed hosts, with a complete loss of variation in the contemporary samples from the Faroe Islands, and an almost complete loss in the sample from Scotland. Although we lack the history of delousing treatments on the farms from which these samples were collected, the presence of only resistant salmon lice (100% resistance according to the T8600C marker) in both the samples suggests that the farms from which these samples were taken have undergone one or several deltamethrin treatments prior to sampling. Alternatively, it could indicate that deltamethrin resistance was so widespread in those geographic regions in that time period, that all sensitive lice, and thus most genetic diversity in mtDNA, was temporarily removed.

Although the contemporary samples representing the Faroese and Scottish sampling areas displayed 100% resistance, it is not possible to rule out that a low frequency of sensitive salmon lice was present in one or both of these regions at the time of sampling or at another time of the year. In contrast to the other contemporary salmon lice collected from farmed hosts, salmon lice collected in Canada appeared to be 100% susceptible (Table 3) and displayed a high mtDNA diversity. This most likely reflects the limited deltamethrin usage in Canada (Fjørtoft et al., 2021), resulting in a reduced selection pressure on the haplotype resistant for deltamethrin. It is important to note that this does not suggest a lack of population genetic connectivity with lice in other regions of the North Atlantic (Besnier et al., 2014; and see discussion below), but is more likely to reflect the “time‐lag” of resistance dispersion coupled with the lack of selection for resistance in that region. The same pattern has been observed for organophosphate resistance (Fjørtoft et al., 2017).

### 4.2 Population genetic structure

Although not a major part of the current study, we found no evidence of population genetic structure among the historical samples for mtDNA nor among the historical or contemporary samples for microsatellites. By contrast, significant population genetic structure was observed for mtDNA among the contemporary samples collected from farmed hosts. However, this was largely driven by the samples collected in the Faroe Islands and Scotland, displaying just one and two haplotypes, respectively, and therefore strongly deviating haplotype frequencies. Neither of those two haplotypes were observed in the historical samples, and both were resistant to pyrethroids according to the marker causatively linked with resistance. Therefore, these observations are consistent with the idea that chemical delousing causes changes in haplotype frequencies, or in this case, selection for the resistant SNP, thus strongly reducing the frequencies of other haplotypes without this SNP. Given the time and spatial scale of this study, these data thus support the results of several previous studies that there is little evidence of stable population genetic structure in this parasite throughout the North Atlantic (Dixon et al., 2004; Glover et al., 2011; Tjensvoll et al., 2006; Todd et al., 2004; Nolan and Powell, 2009).

An earlier study based on analysis of 6000 SNPs throughout the genome concluded that lice were characterized by panmixia in the North Atlantic (Besnier et al., 2014). However, both Besnier et al. (2014) and Jacobs et al. (2018) found evidence of population genetic differentiation in regions of the genome that have subsequently been demonstrated to be linked to emamectin benzoate and organophosphate resistance. Here, we demonstrate this also to be the case for markers linked with pyrethroid resistance. Selection can rapidly alter the allelic frequency of loci under direct selection in the genome and at sites closely linked with them through hitch‐hiking (Kaplan et al., 1989; Smith & Haigh, 2007). We therefore suggest that any observation of population genetic structure in this species, at least in regions of the genome associated with pesticide resistance, is likely to reflect the mosaic of chemical usage in aquaculture in time and space, as opposed to stable population genetic structure created through, for example, limited gene flow.

### 4.3 Multiple origins of deltamethrin resistance in the North Atlantic

In the mitochondrial genes ATPase 6 and Cytochrome B, two haplotypes undetected in the historical samples (haplotypes 165 and 174) dominated in the contemporary samples, suggesting a strong selection for them. Only haplotype 165 was associated with the C14065T marker (section 1.3 in Data S1; Figure S8c). The synonymous C14065T SNP is regarded as a non‐causative mutation (Nilsen & Espedal, 2015). The causative T8600C marker, by contrast, identified both emerging haplotypes 165 and 175 as resistant. Collectively, these data show that the C14065T marker fails to detect resistance status correctly in at least one of the emerging haplotypes, and consequently, results based on this marker (Fjørtoft et al., 2021) only reflect part of the picture of resistance emergence throughout the North Atlantic. This is supported by the fact that haplotype 165 was primarily found in proximity to Norway, where the C14065T marker was identified based on local salmon lice samples. By contrast, haplotype 174 was primarily found in the Faroese sample and occasionally in other mid‐Atlantic locations, with only low frequencies present in the Norwegian samples.

The haplotypes 165 and 174 emerged in the contemporary samples, with haplotype 174 being present in higher frequencies in proximity to the Faroese and haplotype 165 in higher frequencies in proximity to Norway, strongly suggest two primary origins of deltamethrin resistance in the North Atlantic. Another study by Tschesche et al. (2022) supports this interpretation and suggested multiple origins of deltamethrin resistance when studying salmon lice collected in Scotland in 2018–2019. Multiple origins for emergence of pesticide resistance in salmon lice have also been observed for organophosphate resistance located in chromosome 14 (Kaur et al., 2015), where several haplotypes were detected having the causative SNP for organophosphate resistance. By contrast, one main haplotype was found to be under positive selection associated with emamectin benzoate resistance (Besnier et al., 2014) suggesting a primarily single origin of this resistance in the Atlantic.

Although findings of two distinct haplotypes support multiple origins of resistance, it is important to note that new haplotypes associated with resistance may theoretically result from mitochondrial recombination in heteroplasmid individuals without de novo emergence of resistance. Despite being very rare, recombination in mtDNA has been documented in invertebrates (Tsaousis et al., 2005) and may offer an alternative explanation for an origin of the resistant 165 and 174 haplotypes that does not require repetitive emergence of resistance per se. However, our haplotype networks based on the mtDNA genes separately (section 1.3 in Data S1; Figures S6 and S8) are similar to the results from the haplotype network based on the mtDNA genes combined, which suggests that mitochondrial recombination is not likely here.

### 4.4 Dispersal of resistance

The combination of resistant mtDNA haplotypes being present in most of the contemporary samples (Figure 4), the lack of population genetic differentiation indicated by the microsatellite data (Table 4b), and the lack of population genetic differentiation indicated by mtDNA in the historical samples (Table 4a) all point toward to high connectivity among salmon lice in the North Atlantic. Previous studies have also reported a rapid spread of resistant haplotypes and genotypes across the North Atlantic (Besnier et al., 2014; Fjørtoft et al., 2017, 2021) which also bear testimony to a species displaying high genetic connectivity. It may be speculated that the absence of deltamethrin‐resistant lice in the Canadian samples may result from either sampling limitations or a cost associated with resistance that eradicates resistant haplotypes when not under positive selection in that region, or a combination of these factors. In spite of anthropogenic selection evidently eliciting a pattern in the mitochondrial hereditome compartment, the observed arrangement will likely dissipate over time due to mixing and possible negative selection once deltamethrin use is discontinued. Efficient mixing is most likely facilitated by salmon lice attached to wild migrating salmon migrating large distances and meeting their conspecific relatives on common feeding grounds in the North Atlantic (Gilbey et al., 2021). The resulting gene flow will prevent and/or erode the establishment of temporally consistent population structure (Besnier et al., 2014). Hence, the presence of haplotypes 165 and 174 in most of the samples in this study supports the notion of high gene flow in the North Atlantic. The absence of resistance in Canadian samples and the reduced frequencies of resistance in contemporary Norwegian samples compared to Faeroese and Scottish samples suggest that the era of dominance for haplotypes 165 and 174 is likely to crumble with the cessation of deltamethrin usage. This mirrors the values published by Fjørtoft et al. (2021) showing no recorded usage of pyrethroid from year 2000 to 2016 in Canada.

### 4.5 Management implications and future investigation

The parasitic salmon louse, and its widespread resistance to delousing pesticides, provides a persistent challenge to commercial salmonid aquaculture, and represents the largest challenge to sustainable production in the Atlantic (Forseth et al., 2017). Therefore, a thorough understanding of the patterns of emergence and dispersal of resistance is imperative in order to develop informed pest management strategies for the future. Not least, this parasite has repeatedly demonstrated its ability to rapidly adapt to anthropogenic influence such as pesticide usage causing multiresistance (Fjørtoft et al., 2021), altered host parasite dynamics altering life history (Mennerat et al., 2012), non‐chemical delousing treatments on farms potentially leading to changes in pigmentation (Hamre et al., 2021), and salinity and thermal tolerance (Ljungfeldt et al., 2017). In this regard, our data in association with earlier studies demonstrate two important principles: the remarkable adaptive capacity of this parasite and a high degree of connectivity and gene flow. Thus, it follows that there is a great need for amphi‐Atlantic collaboration within management and aquaculture industry to control this parasite.

## Supporting information


Data S1.

Figure S1.

## Data Availability

All data developed during this study are available under supplementary information. An excel file with genotyped resistance data, combined with the mitochondrial sequences for both ATPase 6 and Cytochrome b gene is available under additional file 1. Microsatellite genotypes of the salmon lice used in this study are attached as an excel file under additional file 2. Dryad https://doi.org/10.5061/dryad.2jm63xsvd.
