# Supplementary material for: Aquaculture‐driven evolution of the salmon louse mtDNA genome
Source: Evol Appl. 2023 Jun 28;16(7):1328–44. doi: 10.1111/eva.13572 (PMC10363823; doi:10.1111/eva.13572)
Supplement: Supplementary file 1 — Data S1. [file EVA-16-1328-s002.docx]

**Supplementary file**

- 1. Genetic variation

A total of 263 salmon lice collected from ten locations in the North Atlantic between 2014 and 2017 (contemporary samples) were successfully sequenced for ATPase 6 (A6, 520bp sequenced) and 275 salmon lice successfully sequenced for cytochrome b (Cyt b, 693bp sequenced). Of these sequences 26 sequences for A6 and 65 for Cyt b were single reads only. The successfully sequenced sequences were then aligned to 180 sequences of salmon lice downloaded from GenBank [A6: AY602407 to AY602586, Cyt b: AY602223 to AY602402], originating from six locations in the North Atlantic in the period 2000 to 2002 (historical samples). A total of 196 salmon lice from the contemporary sample and 169 salmon lice from the historical samples were successfully genotyped for 15 microsatellite loci (μSAT).

Standard indices of genetic diversity calculated for all historical and contemporary samples for A6, Cyt b separately (Supplementary Table S1, Supplementary Table S2.). For A6, 263 lice representing the contemporary samples included 107 unique haplotypes (Supplementary Table S1.), with 432 monomorphic nucleotide sites and 88 polymorphic variable sites divided into 44 parsimony-informative (P) and 44 singletons sites among the contemporary samples. For Cyt b, 275 lice representing the contemporary samples included 99 unique haplotypes (Supplementary Table S2.), with 607 monomorphic nucleotide sites and 86 polymorphic variable sites divided into 44 parsimony-informative (P) and 42 singletons sites among the contemporary samples.

For the historical samples, 180 sequences were downloaded from Genebank and trimmed to match the sequence length of 520 base pairs for A6 and 693 base pairs for Cyt b and aligned to the contemporary samples. For A6, 180 lice representing the historical samples included 114 unique haplotypes (Supplementary Table S1.), with 435 monomorphic nucleotide sites and 85 polymorphic variable sites separated into 44 parsimony-informative and 41 singleton variable sites among the historical samples. For Cyt b, 180 lice representing the historical samples included 114 unique haplotypes (Supplementary Table S2.), with 597 monomorphic nucleotide sites and 96 polymorphic variable sites distributed into 50 parsimony-informative (P) and 46 singletons sites among the historical samples.

To account for the uneven sample sizes among the spatial groups, a rarefaction method was used to quantify the haplotype richness (H_Richness_) and the number of private haplotype richness (P_Richness_) within the software program HP-RARE v1.0 (Kalinowski 2005). The minimum sample size was set to 18 individuals for the mitochondrial gene ATPase 6 and 22 individuals for the mitochondrial gene cytochrome b, when comparing the different subsamples. The minimum sample size was set to 180 individuals when comparing between the historical and the contemporary dataset.

For both A6 and Cyt b, the SCO2016 and FAR2016 samples had the lowest measured haplotype richness (H_Richness_) (Supplementary Table S1, Supplementary Table S2). Haplotype richness was significantly higher in the historical (mean A6 = 21.62, mean Cyt b = 22.53) than in the contemporary samples (mean A6 = 13.90, mean Cyt b = 14.61) for both the mitochondrial genes (two sample t-test: A6: H_Richness_: t(10.51) = - 3.32 , p = 0.007; two sample t-test: Cyt b: H_Richness_: t(11.47) = - 3.22, p = 0.008). There was also a significant difference in haplotype richness between the historical (mean A6 = 21.62, mean Cyt b = 22.53) and the contemporary samples (mean A6 = 17.00, mean Cyt b = 17.89) for both genes when excluding the SCO2016 and FAR2016 samples, due to being two outliers having extreme values (two sample t-test: A6: H_Richness_: t(11.18) = - 3.70, p = 0.003; two sample t-test: Cyt b: H_Richness_: t(11.52) = -3.74, p = 0.003).

No private haplotypes (P_Richness_) were detected within the SCO2016 and FAR2016 samples when adjusted for the minimum sample size for both the mitochondrial genes (Supplementary Table S1, Supplementary Table S2.). Private haplotype richness was significantly different between the historical (mean A6= 11.12, mean Cyt b = 13.88) and the contemporary (mean A6 = 6.38, mean Cyt b = 6.72) samples for both the mitochondrial genes (two sample t-test: A6: P_Richness_: t(10.62) = -3.39, p = 0.006; two sample t-test: Cyt b: P_Richness_: t(10.49) = - 3.57, p = 0.005). There was also a significant difference in private haplotype richness between the historical (mean A6 = 11.12, mean Cyt b = 13.88) and the contemporary (mean A6 = 7.97, mean Cyt b = 8.40) samples for both genes when excluding the SCO2016 and FAR2016 sample, again due to being two outliers (two sample t-test: A6: P_Richness_: t(9.12) = -2.86, p = 0.018; two sample t-test: Cyt b: P_Richness_: t(6.38) = -3.21, p = 0.017).

**Supplementary Table S1**. Summary of mtDNA haplotype diversity (ATPase 6) for the historical and contemporary samples. The table shows the host species for the collected salmon lice (WS- wild salmon, WT- wild seatrout, FS- farmed salmon), sample size (N), the observed number of haplotypes (N_Haplotypes_), haplotype richness (H_Richness_) and private haplotype richness (P_Richness_). The table also shows haplotype diversity (H_Diversity_) with standard deviations in brackets, nucleotide diversity (π) and the number of SNPs (N_SNP_) in each sample.

**Supplementary Table S2.** Summary of mtDNA haplotype diversity (cytochrome b) for the historical and contemporary samples. The table shows the host species for the collected salmon lice (WS- wild salmon, WT- wild seatrout, FS- farmed salmon), sample size (N), the observed number of haplotypes (N_Haplotypes_), haplotype richness (H_Richness_) and private haplotype richness (P_Richness_). The table also shows haplotype diversity (H_Diversity_) with standard deviations in brackets, nucleotide diversity (π) and the number of SNPs (N_SNP_) in each sample.

- 1. Population genetic structure

To investigate spatial and temporal genetic structure among samples, pairwise comparisons were conducted for the two mtDNA genes separately to account for possible recombination (Supplementary Table S3, Supplementary Table S4).

For A6 and Cyt b, no significant genetic differentiation was observed among the historical samples (Supplementary Table S3, Supplementary Table S4). However, among the contemporary samples, significant genetic differentiation was observed. In particular, the FAR2016 and SCO2016 samples deviated significantly from each other and from all other samples, with pairwise values ranging from 0.303 to 0.520 for A6 and 0.341 to 0.606 for Cyt b in FAR2016 and from 0.014 to 0.195 for A6 and 0.046 to 0.236 for Cyt b in SCO2016. It is important to note that both samples comprised only one (FAR2016) and two haplotypes (SCO2016), which is the reason for the substantial genetic differentiation observed.

There were significant deviations in haplotype frequencies between some of the contemporary and historical samples. Notably, the FAR2016 and SCO2016 samples differed significantly from the historical samples. The contemporary FAR2016 sample differed from Φst 0.397 (pair-wise comparison with CAN2002) to Φst 0.453 (pair-wise comparison with SCO2002) for A6, and from Φst 0.500 (pair-wise comparison to NO(S)2002) to 0.581 (pair-wise comparison with NO(W)2002) for Cyt b. The contemporary SCO2016 sample differed from Φst 0.159 (pair-wise comparison with RUS2000) to Φst 0.193 (pair-wise comparison with NO(N)2000) for A6, and from Φst 0.192 (pair-wise comparison with NO(S)2002) to 0.232 (pair-wise comparison with CAN2002) for Cyt b.

**Supplementary Table S3.** Pairwise genetic differences among historical and contemporary samples of lice using mtDNA ATPase 6 (Φ_ST_ in the lower diagonal, Holm-Bonferroni sequential corrected p-value in the upper right diagonal). Significant values < 0.05 are highlighted in bold. The color gradient shows the level of differentiation as suggested by Wright (1978). Green being low differentiation (< 0.05), yellow intermediate (0.05-0.15), and red high differentiation (> 0.15).

**Supplementary Table S4.** Pairwise genetic differences among historical and contemporary samples of lice using mtDNA cytochrome b (Φ_ST_ in the lower diagonal, Holm-Bonferroni sequential corrected p-value in the upper right diagonal). Significant values < 0.05 are highlighted in bold. The color gradient shows the level of differentiation as suggested by Wright (1978). Green being low differentiation (< 0.05), yellow intermediate (0.05-0.15), and red high differentiation (> 0.15).

- 1. Haplotype distribution

Haplotype networks was generated separately for A6 and Cyt b using the Medium Joint Network approach. The first striking observation from the Cyt b networks is that two haplotypes, C118 and C126 (C indicates that it is a Cyt b haplotype and the following number refers to the haplotype number) did not exist in the historical samples but emerged as the two most frequent haplotypes in the contemporary samples (Supplementary Fig. S2). Of the total 275 salmon lice within the contemporary Cyt b sample, 47 and 59 displayed haplotype C118 and C126, respectively.

**Supplementary Fig. S2.** Haplotype network of the historical (blue) and contemporary (pink) samples of salmon lice (cytochrome b). Note the emergence of two haplotypes within the contemporary sample (Hap C118 and Hap C126). Nodes represent one haplotype. The size and the color within each node correspond to the number of sequences per group that shares the haplotype. The number of homologous nucleotide sites where two haplotypes differ is represented by the number of parallel lines. 0 to 1 parallel line represents one mutational difference.

In the contemporary Cyt b sample, pyrethroid resistant (linked to mtDNA) salmon lice were only found among four haplotypes (Supplementary Fig. S3 b/c). When looking further at the relationship between pyrethroid resistance and haplotype it becomes clear that most individuals with haplotype C118 were resistant and that most of the resistant lice displayed haplotype C118 (Supplementary Fig. S3 c). Of the resistant lice, all samples except NO(S)2014, FAR2016, and CAN2017 which did not comprise resistant salmon lice according to the C14065T marker, were represented within haplotype C118 (Supplementary Fig. S3 b). Whereas all samples, except CAN2017, had salmon lice comprising haplotype C126 (Supplementary Fig. S3 a). However, contrastingly to haplotype C118, the majority (93%) displaying haplotype C126 were marked as sensitive (Supplementary Fig. S3 c).

The highest percentage of pyrethroid resistant salmon lice (47 %) was found in the SCO2016 sample, which comprised only haplotypes C118 and C126 (Supplementary Fig. S3 a/ b.). Of the 32 lice belonging to the SCO2016 sample 13 were haplotype C118 and 12 of these were pyrethroid resistant. The remaining 19 lice from this sample were haplotype C126 and 16 of these were sensitive to pyrethroids. All lice within the FAR2016 sample displayed haplotype C126 (Supplementary Fig. S3 a.), and notably, all were susceptible to pyrethroid.

**Supplementary Fig. S3 a-c.** Haplotype network of the contemporary samples of salmon lice (cytochrome b). a) Network of sensitive (S) individuals, and b) Network of resistant (R) individuals for deltamethrin based upon the C14065T genetic marker separated by sample location. Sample location in Fig. a) marked with (*) are samples comprising resistant individuals in Fig. b). c) Network separated into sensitive (S) and resistant individuals (R) for deltamethrin based upon the C14065T genetic marker. The size and the color within the nodes correspond to the number of sequences per group that share a haplotype. The number of homologous nucleotide sites where two haplotypes differ is represented by the number of parallel lines. 0 to 1 parallel line represents one mutational difference. The figure shows the emergence of two haplotypes (Hap C118 and Hap C126), with the most pyrethroid-resistant individuals displaying Hap C118.

The same patterns for haplotype distribution for Cyt b were seen in the mitochondrial gene A6 for both the historical and the contemporary samples (Supplementary Fig. S5.), with two emerged haplotypes in the contemporary sample (A7 and A124, where A indicates that it is a A6 haplotype and the following number refers to the haplotype number). The A7 haplotype dominate among the pyrethroid resistant individuals being found in 49 of the total 54 individuals (Supplementary Fig. S6 b.). However, in contrast to Cyt b, one of the two emerged haplotypes (haplotype A7) was present in the historical sample also.

For the contemporary Cyt b dataset, haplotype C126 was mainly dominated around the Faroe Islands area. There were also an increased number of individuals with this haplotype in Scotland and Ireland (Supplementary Fig. S4.). Haplotype C118 has been found with increased frequency along the Norwegian coastline, except for the southern-eastern part of Norway. At all sample locations, either both or one of the emerging haplotypes (haplotype C118 and haplotype C126) were found, except for the sample collected on the eastern coast of Canada (CAN2017). The same tendency is observed within the mitochondrial gene A6. However, haplotype A7 was also found within the CAN2017 sample for A6 (Supplementary Fig. S7.).


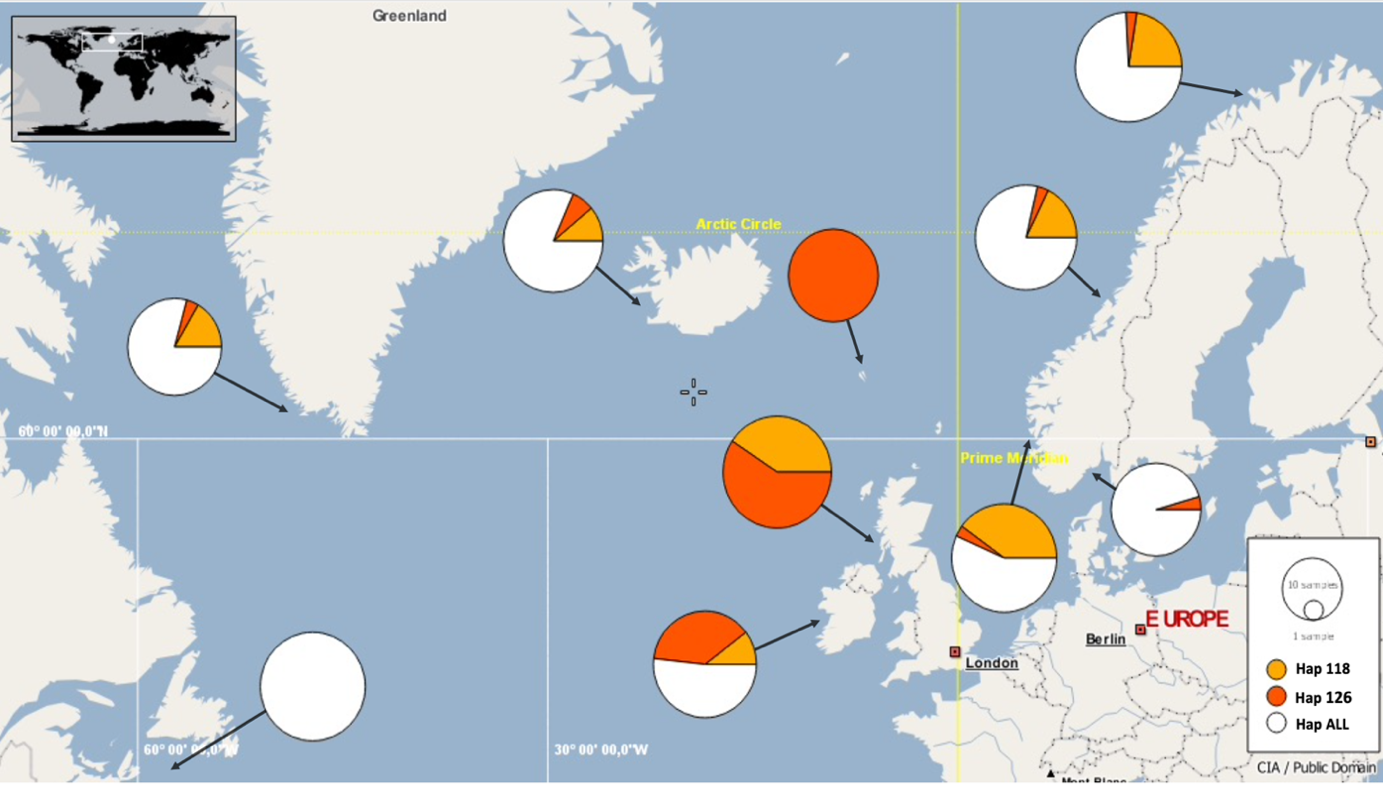


**Supplementary Fig. S4.** Map of North Atlantic, showing the distribution of the two emerged haplotypes for the mitochondrial gene cytochrome b (Hap 118 and Hap126), relative to the rest of the observed haplotypes (Hap ALL) merged into one pie of the contemporary lice.

**Supplementary Fig. S5.** Haplotype network of the historical (blue) and contemporary (pink) samples of salmon lice (ATPase 6). Note the emergence of two haplotypes within the contemporary sample (Hap A7 and Hap A124). Nodes represent one haplotype. The size and the color within each node correspond to the number of sequences per group that shares the haplotype. The number of homologous nucleotide sites where two haplotypes differ is represented by the number of parallel lines. 0 to 1 parallel line represents one mutational difference.

****Supplementary Fig. S6 a-c.** Haplotype network of the contemporary samples of salmon lice (ATPase 6). a) Network of sensitive (S) individuals, and b) Network of resistant (R) individuals for deltamethrin based upon the C14065T genetic marker separated by sample location. Sample location in Fig. a) marked with (*) are samples comprising resistant individuals in Fig. b). c) Network separated into sensitive (S) and resistant individuals (R) for deltamethrin based upon the C14065T genetic marker. The size and the color within the nodes correspond to the number of sequences per group that share a haplotype. The number of homologous nucleotide sites where two haplotypes differ is represented by the number of parallel lines. 0 to 1 parallel line represents one mutational difference. The figure shows the emergence of two haplotypes (Hap A7 and Hap A124), with the most pyrethroid-resistant individuals displaying Hap A7.

*
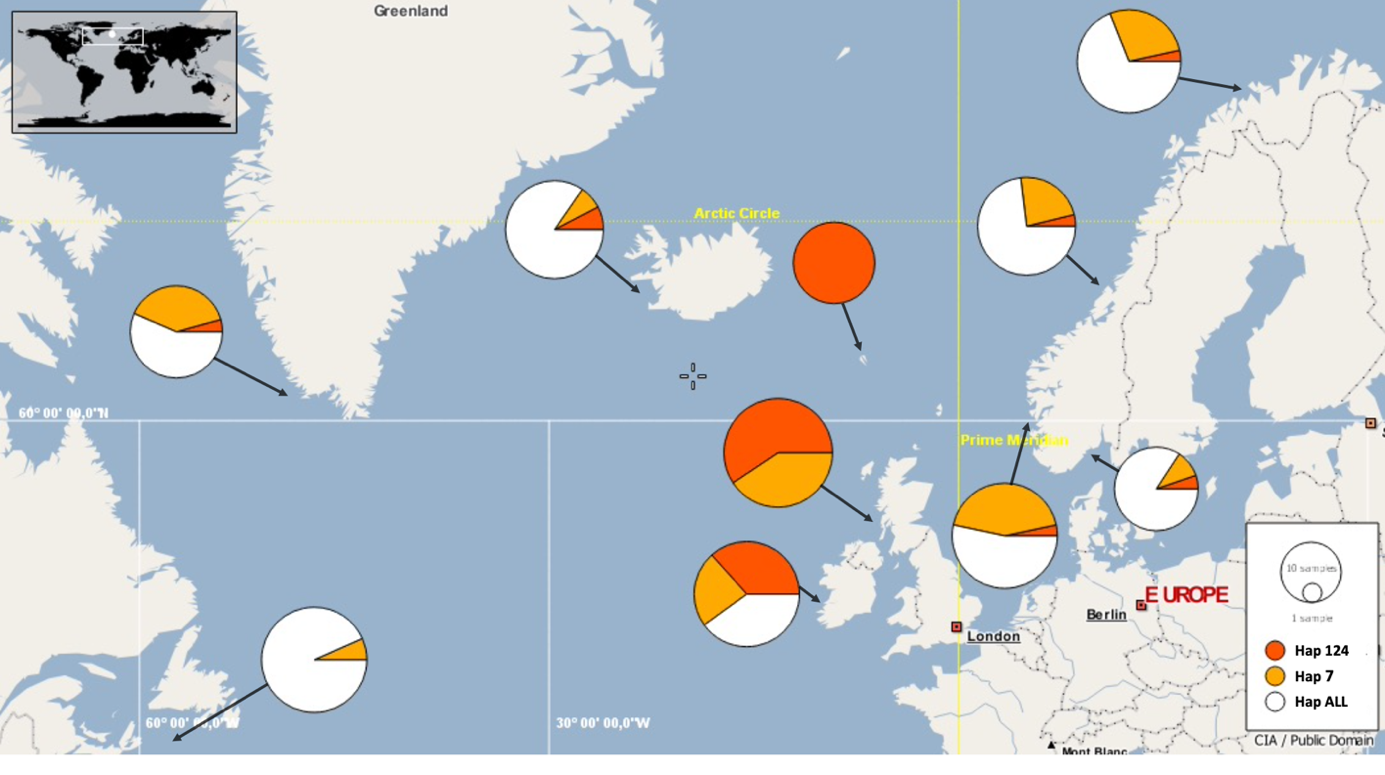
***Supplementary Fig. S7.** Map of North Atlantic, showing the distribution of the two emerged haplotypes for the mitochondrial gene ATPase 6 (Hap 7 and Hap 124), relative to the rest of the observed haplotypes (Hap ALL) merged into one pie of the contemporary lice.

**Supplementary Fig. S8 a-c.** Haplotype network of the contemporary samples of salmon lice (haplotypes created across cytochrome b and ATPase 6). a) Network of sensitive (S) individuals, and b) Network of resistant (R) individuals for deltamethrin based upon the C14065T genetic marker separated by sample location. Sample location in Fig. a) marked with (*) are samples comprising resistant individuals in Fig. b). c) Network separated into sensitive (S) and resistant individuals (R) for deltamethrin based upon the C14065T genetic marker. The size and the color within the nodes correspond to the number of sequences per group that share a haplotype. The number of homologous nucleotide sites where two haplotypes differ is represented by the number of parallel lines. 0 to 1 parallel line represents one mutational difference. The figure shows the emergence of two haplotypes (Hap 165 and Hap 174), with the most pyrethroid-resistant individuals displaying Hap 165.
