# Supplementary figures and images for: Aquaculture‐driven evolution of the salmon louse mtDNA genome
Source: Evol Appl. 2023 Jun 28;16(7):1328–44. doi: 10.1111/eva.13572 (PMC10363823; doi:10.1111/eva.13572)

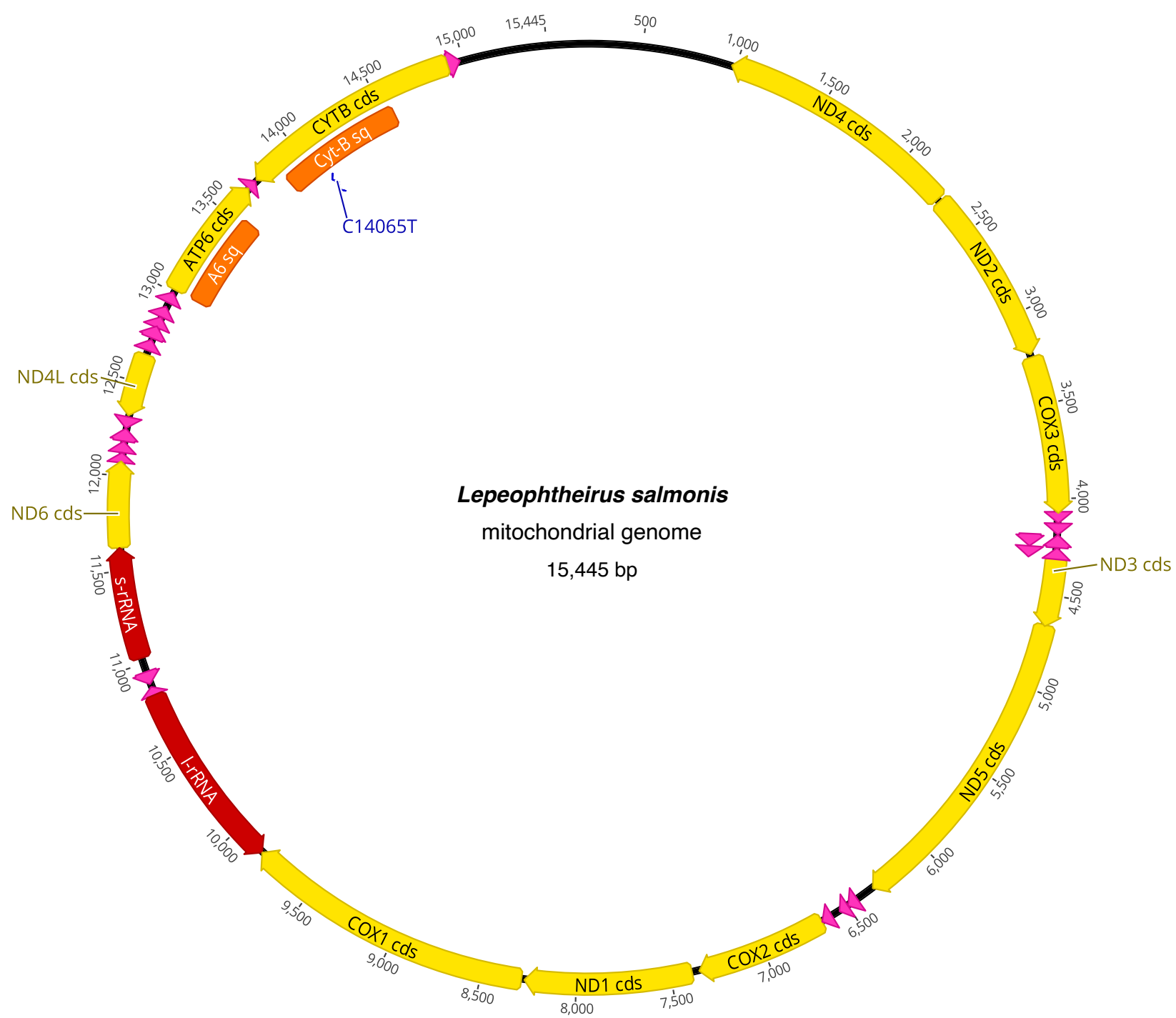

Supplement: Supplementary file 2 — Figure S1. [file EVA-16-1328-s001.pdf]
